# Aquatic moss precipitates metals in the presence of a specific endophytic microbiome

**DOI:** 10.1002/imo2.70061

**Published:** 2025-10-21

**Authors:** Kaisa Lehosmaa, Janne J. Koskimäki, Olga Podolich, Saija H. K. Ahonen, Anna Liisa Ruotsalainen, Piippa R. Wäli, Maria Greger, Urooj Rashid, Namrata Baruah, Jaanika Edesi, Annamari Markkola, Sepideh Kiani, Heini Postila, Katharina Kujala, Anna‐Kaisa Ronkanen, Anna Maria Pirttilä

**Affiliations:** ^1^ University of Oulu, Ecology and Genetics Research Unit Oulu Finland; ^2^ Institute of Molecular Biology and Genetics NASU, Kyiv Kyiv Ukraine; ^3^ Department of Ecology Stockholm University Stockholm Sweden; ^4^ Centre of Estonian Rural Research and Knowledge METK Jõgeva alevik Estonia; ^5^ Boliden Kevitsa Mining Oy Petkula Finland; ^6^ University of Oulu, Water, Energy and Environmental Engineering Research Unit Oulu Finland; ^7^ Natural Resources Institute Finland (LUKE) Oulu Finland; ^8^ Finnish Environment Institute Marine and Freshwater Solutions Oulu Finland

## Abstract

Harnessing moss–microbe interactions for metal removal. Schematic representation of aquatic moss *Warnstorfia fluitans* and its microbial symbionts in the remediation of metal‐contaminated waters. Amplicon sequencing and advanced microscopy revealed microbial community composition and role of moss symbionts in metal accumulation within *the moss* tissues from mining‐impacted and control sites. The moss efficiently accumulates metals, including iron, which is stored intracellularly near the cell membrane in association with endophytic microorganisms, and mosses in metal‐rich environments host a distinct microbiome with reduced diversity compared to control. In metal‐rich conditions, endophytic fungal species *Phialocephala bamuru* and *Hyaloscypha hepaticola* were abundant and cultivated, potentially contributing to metal tolerance or accumulation in the moss. These results suggest that *W. fluitans* along with its metal‐tolerant microbial symbionts offers exciting potential for innovative nature‐based purification systems for metal‐contaminated waters.

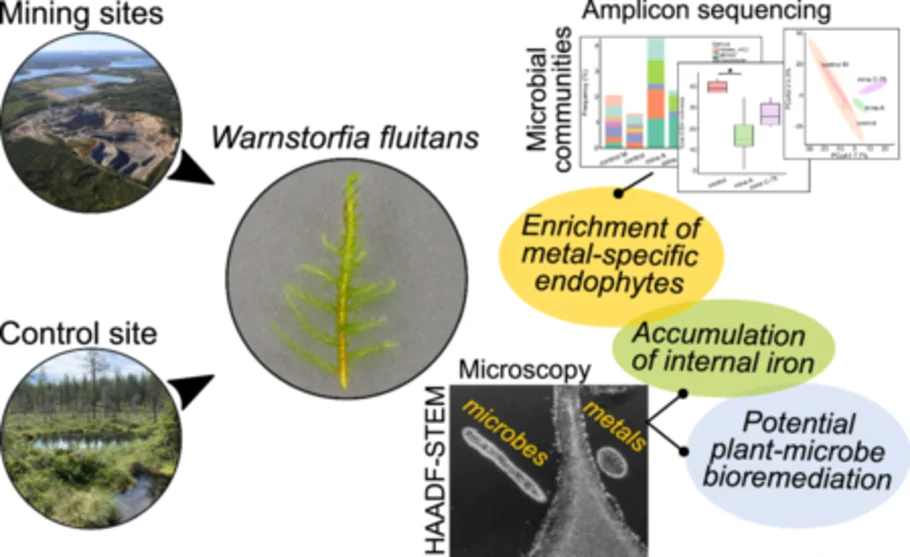

## AUTHOR CONTRIBUTIONS

**Kaisa Lehosmaa**: Designing & data collection; surface sterilization & molecular analyses; cultivation; TEM microscopy; formal analyses; visualization; writing—original draft; methodology; writing—review & editing. **Janne J. Koskimäki**: Surface sterilization & molecular analyses; visualization; writing—review & editing. **Olga Podolich**: TEM Microscopy; writing—review & editing. **Saija H. K. Ahonen**: Surface sterilization & molecular analyses; cultivation; formal analyses; writing—review & editing. **Anna Liisa Ruotsalainen**: Designing & data collection; writing—review & editing. **Piippa R. Wäli**: designing & data collection; writing—review & editing. **Maria Greger**: Formal analyses. **Urooj Rashid**: Cultivation. **Namrata Baruah**: Surface sterilization & molecular analyses; cultivation. **Jaanika Edesi**: Surface sterilization & molecular analyses. **Annamari Markkola**: Designing & data collection; writing—review & editing. **Sepideh Kiani**: Designing & data collection. **Heini Postila**: Designing & data collection. **Katharina Kujala**: Designing & data collection; writing—review & editing. **Anna‐Kaisa Ronkanen**: Designing & data collection; writing—review & editing. **Anna Maria Pirttilä**: Designing & data collection; visualization; writing—original draft; methodology; writing—review & editing. All authors have read the final manuscript and approved it for publication.

## CONFLICT OF INTEREST STATEMENT

The authors declare no conflicts of interest.

## ETHICS STATEMENT

No animals or humans were involved in this study.

To the Editor,

Due to mining expansion in high‐latitude regions, anthropogenic pressure has intensified the input of metals into aquatic ecosystems. Such sensitive environments require effective, sustainable remediation strategies suitable for cold climate conditions [1]. In general, plants in association with microorganisms can clean contaminated soils and water in a process called phytoremediation [2]. Plant‐associated microorganisms, endophytes living inside plant tissues, and epiphytes found on plant surfaces, play vital roles in enhancing plant stress tolerance and detoxification capabilities [3, 4]. In metal‐rich environments, these microbial symbionts can modulate metal bioavailability, assist in metal uptake or sequestration, and contribute to host resilience via enzymatic detoxification and redox reactions [4, 5]. Aquatic bryophytes, such as mosses, are especially valuable in remediation processes due to their high surface area, capacity for direct element uptake through cell walls, and adaptation to low‐nutrient, cold, and acidic environments [6, 7]. *Warnstorfia fluitans*, a submerged aquatic moss common in subarctic and Arctic freshwater systems, is such a candidate. Previous studies have shown that *W. fluitans* can absorb arsenic and nitrogen from contaminated waters [6, 7, 8], but the role of microbial symbionts and potential for metal remediation remain largely unexplored. We investigated the microbial communities of *W. fluitans* in metal‐rich and control environments. Using amplicon sequencing and advanced microscopy, we characterized the diversity and spatial localization of moss microbiome, assessed metal accumulation patterns within moss tissues, and isolated dominant fungal taxa from metal‐rich sites. *W. fluitans* efficiently accumulated metals, particularly Fe, which was stored intracellularly near the cell membrane in association with endophytic microorganisms. Furthermore, mosses from metal‐rich sites hosted a distinct and less diverse microbiome, specifically endophytic fungi. Fungal endophytes *Phialocephala bamuru* and *Hyaloscypha hepaticola* were identified as candidates for contributing to metal tolerance or sequestration in floating‐hook moss. Our findings suggest that *W. fluitans*, along with its metal‐adapted microbial symbionts, forms a functional holobiont with promising potential for use in nature‐based purification systems designed for cold, metal‐contaminated waters.

## 1 Moss sampling

*W. fluitans* (Figure 1A,B) was sampled in September 2018 from one active and two closed mining sites (Figure S1), as well as one control site in the subarctic climate zone. Selection of the sites was based on the natural occurrence of *W. fluitans*, which reflects its long‐term ecological adaptation to various environments. The active mining site was the deepest base metal mine in Europe, located near Pyhäjärvi, central Finland, hereafter referred to as mine A. The two closed mines were a copper mine, which ceased in 1978, in Adakgruvan, Sweden, hereafter mine C‐78, and the gold mine Saattopora, which is in Finnish Lapland and closed in 1996, hereafter mine C‐96. The control site was a groundwater‐dependent spring ecosystem located in Finnish Lapland, hereafter referred to as the control.

**FIGURE 1:**
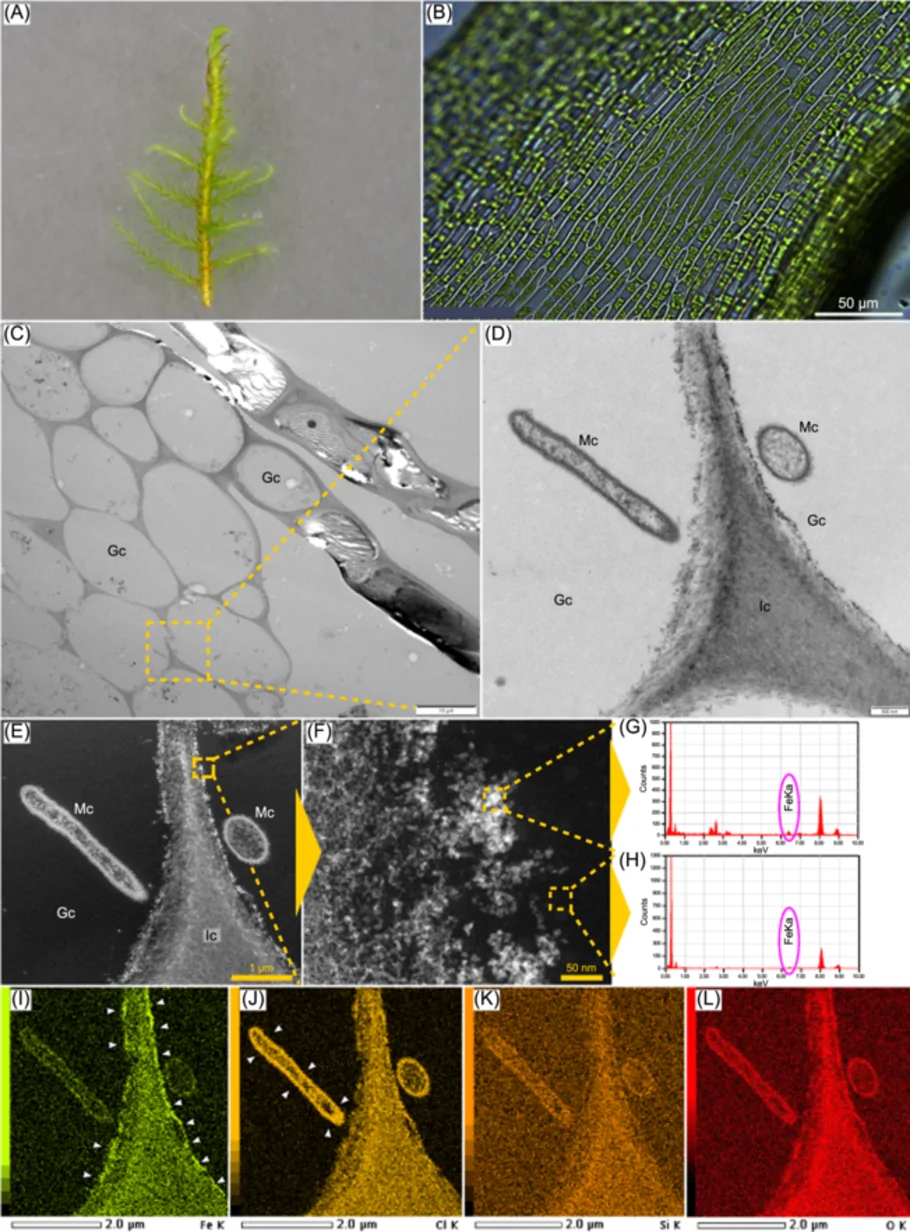
Visualization of *Warnstorfia fluitans* and associated microbes through light and transmission electron microscopy (TEM). (A) A shoot of *W. fluitans*. (B) Light microscopy image of a longitudinal section of *W. fluitans* shoot. TEM (C, D) and selected area electron diffraction (HAADF‐STEM) (E, F) images of moss tissue from mine A (uranyl acetate and lead citrate stained sample), with (G, H) line scan images of the precipitated elements within the cell (G, Fe particles localized along inner cell wall and H, displays a background signal with low Fe presence). HAADF‐STEM detected elements (I–L), including iron (I), chloride (J), silica (K), and oxygen (L). Abbreviations in Figure C–E are, Mc = microbial cell, Gc = gametophyte cell, and Ic = intercellular space. Dashed squares indicate sites of magnification (C, E) or line scanning (F). Scale bars (B) 50 μm, (C) 10 μm, (D) 500 nm, (E) 1 μm, (F) 50 nm, and (I–L) 2 μm.

## 2 Metal accumulation in moss tissue

The highest metal concentrations were found in the moss tissue from the sites of mine A and mine C‐96, whereas the samples from mine C‐78 had low metal quantities (Table S1). Specifically, the quantities of Fe, Cd, Cu, Zn, Ni, and As were significantly higher (*t*‐test; *p* < 0.05) in mine A compared to the control. The highest concentrations of As were found in the closed site (mine C‐96) (Table S1), which is situated in the Western Lapland of Finland, an area recognized for its greenstone belt. The mine C‐96, previously a gold mine, is notable for its arsenopyrite (FeAsS) deposits, which have resulted in arsenic‐rich wastewater. Aquatic mosses absorb water through their outer surface, allowing metals to enter their tissues [6].

According to transmission electron microscopy of moss tissue, the elements, particularly iron and chloride, were precipitated in the moss tissue, intracellularly close to the cell membrane (Figure 1C,D). We also found microorganisms inside the moss gametophyte cells associated with iron (1.66 mass%) and chloride (4.0 mass%) (Figure 1D−L). Gametophyte cells play prominent roles not only in plant‐microbe interactions, as noted by [9], but also in creating conditions suitable for iron precipitation, such as elevated pH, similar to the hyaline cells of *Sphagnum* [10]. The endophytic microorganisms that we localized in the presence of metals could be responsible for facilitating metal uptake or enhancing plant tolerance to metals [4]. According to selected area electron diffraction, the iron was in an amorphous form inside the cells, attached to the cell membrane. The compound was associated with both chloride and oxygen in a non‐ionic form (Figure 1G,I,J,L, and Figure S2). Mine A had a high iron concentration in the incoming water (Table S2), which is consistent with iron accumulation in the moss cells. The precipitated metals were not observed in the moss tissues of the control site (Figure S3). The results were based on comparisons of stained (uranyl acetate and lead citrate; Figure 1C) and unstained samples (Figure S2) to detect both microorganisms and metals simultaneously.

The mechanisms of metal uptake have mainly been studied in plant‐associated bacteria of terrestrial plants. In land plants, metal uptake is enhanced by bacterial production of organic acids and siderophores, as highlighted by [11]. Metals, particularly iron, can undergo intracellular precipitation aided by bacteria via active biosorption, a process documented by [12]. The active biosorption has earlier been detected in bacterial endophytes of hyperaccumulator plants [4]. A similar mechanism has also been observed in mycorrhizal fungi [13]. There is less evidence on fungal endophytes, but genera such as *Penicillium*, *Aspergillus*, and *Trichoderma*, have been reported to secrete metabolites or extracellular polymeric substances that can bind metal ions such as Cd and Ni, leading to their precipitation or immobilization [14].

## 3 Moss microbiome

The mosses harbored a specialized endophytic mycobiome at each metal‐rich mining site, as the endophyte assemblages significantly differed in the mining sites from the control (PERMANOVA *p* < 0.01; Figure 2A). A similar pattern was observed for the full mycobiome composition (comprising both epi‐ and endophytic fungi) (*p* = 0.001; Figure 2B) and for the full moss bacteriome (*p* = 0.001; Figure 2C). The microbiome of aquatic mosses is derived from planktonic microorganisms present in the water, which originate from soil communities of the riparian zone [15]. The studied mining sites differed based on the mined ore, resulting in variations in water pH, metal concentrations, and other environmental factors. These factors can influence the long‐term genetic adaptation and speciation of the moss microbiome [3]. Endophytic communities are dynamic, undergoing transformations during plant development, and the host plant can influence their structure [16]. In metal‐rich, acidic environments, plant microbiomes, such as fungal endophytes, can provide additional genes that aid the host plant in adapting to the local conditions [5].

**FIGURE 2:**
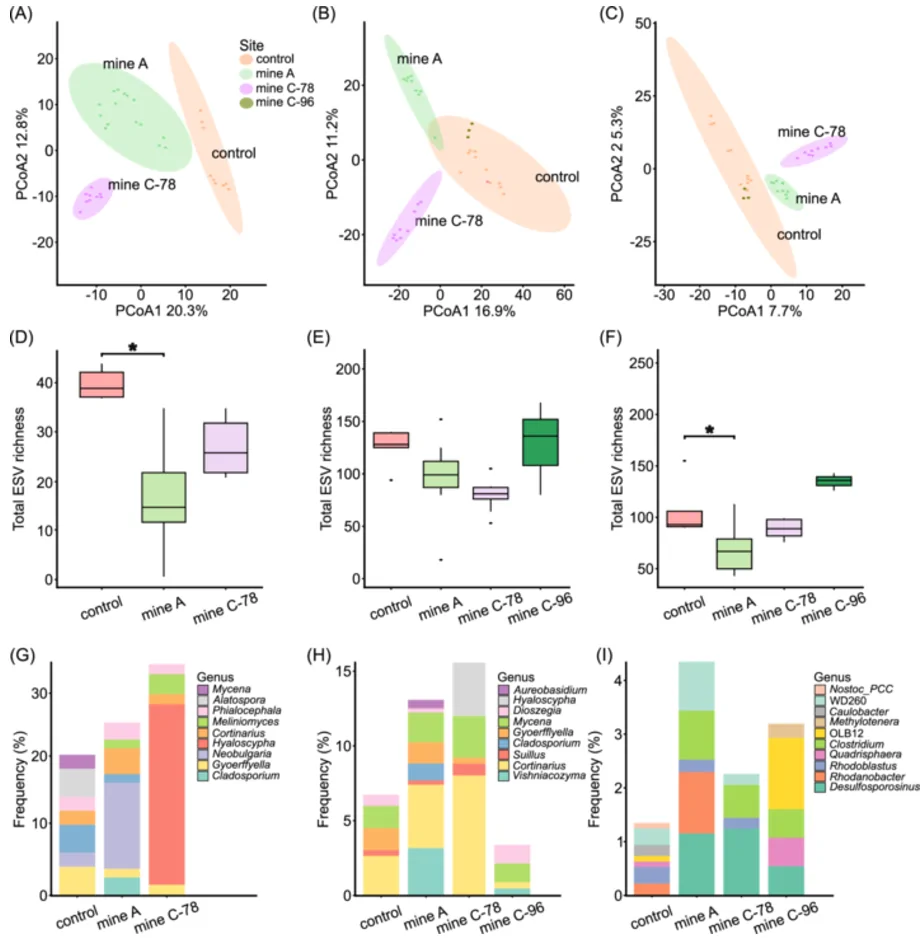
Characterization of *Warnstorfia fluitans* microbial symbionts. Principal coordinate analysis (PCoA) ordination of fungal endophyte (A), mycobiome (B), and bacteriome composition (C) among control and mining sites. Ellipses are 95% Confidence Intervals around group centroids. Boxplots in D–F represent median values with interquartile range of ESVs (exact sequence variants) observed fungal endophyte (D), mycobiome (E), and bacteriome richness (F). All comparisons are made against the control; significant differences are denoted by asterisks (* *p* < 0.05). Figure G–I are core genera i.e., abundance bar plots for nine (G, H) and ten (I) most abundant genera of fungal endophyte (G), mycobiome (H), and bacteriome (I) data. Control = site with relatively low metal concentrations, mine A = active mine, and mines C‐78 and C‐96 are closed mines.

We observed significant variations in the diversity of microorganisms living in symbiosis with moss across the different sites. Specifically, the control site demonstrated higher fungal endophyte diversity than the mining sites, particularly when compared to the active mine A (*p* < 0.05; Figure 2D). This suggests that low water pH and elevated metal concentrations reduce the diversity of the fungal endosphere, while in the full moss mycobiome, variations in the diversity between control and mining sites were less distinct (Figure 2E). The active mining site (mine A) also exhibited a lower bacterial diversity compared to the control (*p* = 0.04; Figure 2F). The pH, which was the lowest at the active mining site, can be responsible for driving the bacterial diversity in *W*. *fluitans*, as the active mining site was extremely acidic compared to the other sites. Furthermore, bacteria tend to have narrower and specific pH optima for growth compared to fungi. In the closed mines, especially in mine C‐96, the bacterial richness resembled more that of the control (Figure 2F).

## 4 Moss symbionts at different sites

Amplicon sequencing of microbial communities revealed distinct compositional shifts between control and mining sites, with several taxa showing site‐specific enrichment. Notably, endophytic members of the order *Helotiales* were differentially abundant at the mining sites, particularly the species *Hyaloscypha hepaticola* and *Phialocephala bamuru* (Table S3). These two taxa were also successfully isolated as fungal endophytes from mosses growing at the active mining site A (*P. bamuru*) and the closed mining site C‐78 (*H. hepaticola*) where it was also highly abundant (Figure 2G). An internal transcribed spacer sequence analysis of the isolates confirmed their identity with 99% similarity to reference sequences, and they were not detected in other study sites, reinforcing their association with the mining‐impacted environments. *H. hepaticola* is a mycorrhizal fungus that forms intracellular associations with several leafy liverworts [17]. It is widely found in ericaceous plants, which require low pH conditions, similar to mosses, and earlier, moss rhizoids have been suggested to play a role in the formation of ericoid mycorrhizas [17]. Our study is the first where *H. hepaticola* was identified abundant, being isolated from an aquatic moss without a mycorrhizal association. In terrestrial ecosystems, ericoid mycorrhizal fungi have been reported to increase plant tolerance to toxic metals and detoxify soil [18]. In ericoid dwarf shrubs, the root‐associated *H. hepaticola* has been found to improve plant tolerance to Fe and to reduce foliar Fe and Mn [19]. *P. bamuru* is a dark septate endophyte (DSE) previously isolated from the roots of herbaceous plants. DSEs show no host specificity and are found in stressful abiotic conditions, such as metal‐rich soils [20]. Our study is the first one reporting *P. bamuru* in symbiosis with floating hook‐moss, and moss tissue rich in metals. Additional mycobiome taxa enriched at mining sites included *Cladosporium cladosporioides*, *Hypogymnia physodes*, *Botrytis cinerea*, and *Paraphaeosphaeria angularis*, with *Cladosporium* and *Cortinarius* being particularly frequent and identified as indicator taxa for active mining site (Figure 2H). Other endophytic taxa associated with mining sites included *Neobulgaria sp*., *Meliniomyces sp*., and *Gyoerffyella entomobryoides*, the latter also appearing in the control site. Overall, the mosses from the control site were associated with distinct fungal communities, characterized by the presence of *Aureobasidium pullulans*, *Lemonniera centrosphaera*, and *Sanchytrium sp*. Bacterial taxa were not differentially abundant, although certain genera, such as *Desulfosporosinus*, *Clostridium*, *Rhodanobacter*, and Pseudomonadota group WD260, earlier found in northern peatlands, were more abundant in mining sites, particularly at mine A (Figure 2I). In our study, endophytic fungi stood out as pivotal taxa uniquely adapted to thrive amidst metal‐rich environments. Overall, attention paid to fungi in bioremediation is extremely low and should be increased, because they play central roles in the biochemical cycles of elements in the aquatic systems. Our results demonstrate that *W. fluitans* together with its microbial symbionts, such as endophytic fungi, possesses promising potential for use in nature‐based purification systems in cold, metal‐rich conditions.

## Supporting information


**Figure S1.** Map of sampling sites in Finland and Sweden.

**Figure S2.** Transmission electron microscopy (TEM) of an unstained mine A sample.
**Figure S3.** Transmission electron microscopy (TEM) of a stained and unstained control sample.

## Data Availability

The data that support the findings of this study are available in the supplementary material of this article. Sequence data is deposited under SRA Archive accession number PRJNA1106245 in the NCBI BioProject database (https://www.ncbi.nlm.nih.gov/sra/?term=PRJNA1106245). The data and scripts used are saved in GitHub https://github.com/Lehosmaa/Moss-microbe-metal-interactions.git. Supplementary materials (methods, figures, tables, graphical abstract, and update materials) may be found in the online DOI or iMetaOmics http://www.imeta.science/imetaomics/.
