# Supplementary material for: Aquatic moss precipitates metals in the presence of a specific endophytic microbiome
Source: IMetaOmics. 2025 Oct 21;3(1):e70061. doi: 10.1002/imo2.70061 (PMC13674383; doi:10.1002/imo2.70061)
Supplement: Supplementary file 1 [file IMO2-3-e70061-s001.docx]

Supporting information to

**Aquatic moss precipitates metals in the presence of a specific endophytic microbiome**

**Running title**: Microbially mediated metal precipitation in aquatic moss

Kaisa Lehosmaa^1^*, Janne J. Koskimäki^1^, Olga Podolich^1,2^, Saija H.K. Ahonen^1^, Anna Liisa Ruotsalainen^1^, Piippa R. Wäli^1^, Maria Greger^3^, Urooj Rashid^1^, Namrata Baruah^1^, Jaanika Edesi^4^, Annamari Markkola^1^, Sepideh Kiani^5,6^, Heini Postila^6^, Katharina Kujala^7^, Anna-Kaisa Ronkanen^8^, Anna Maria Pirttilä^1^

^1^University of Oulu, Ecology and Genetics Research Unit, Oulu, FI-90014, Finland

^2^ Institute of Molecular Biology and Genetics NASU, Kyiv, 03143, Ukraine

^3^ Stockholm University, Department of Ecology, Environment and Plant Sciences, Stockholm, SE-10691, Sweden

^4^ Centre of Estonian Rural Research and Knowledge METK, Jõgeva alevik, 438309, Estonia

^5^ Boliden Kevitsa Mining Oy, Petkula, FI-99670, Finland

^6^ University of Oulu, Water, Energy and Environmental Engineering Research Unit, Oulu, FI-90014, Finland

^7^ Natural Resources Institute Finland (LUKE), Oulu, FI-90570, Finland

^8^ Finnish Environment Institute, Marine and freshwater solutions, Oulu, FI-90570, Finland

*Correspondence: [Kaisa.Lehosmaa@oulu.fi](mailto:Kaisa.Lehosmaa@oulu.fi) (Kaisa Lehosmaa)

**METHODS**

**Sampling sites**

For the microbiome analysis, the *W.fluitans* was sampled in September 2018 from one active and two closed mining sites, as well as one control site in the subarctic climate zone. Sampling sites were chosen according to human impact and land use, controls representing pristine sites. The selection of these sites was based on the natural occurrence of the *W.fluitans*, present in active and closed mining sites and control site, which reflects its long-term ecological adaptation to various environments. The active mining site was the deepest base metal mine in Europe, located near Pyhäjärvi, central Finland (63°40’N; 25°58’E; hereafter referred to as mine A). The two closed mines were a copper mine, which ceased in 1978, in Adakgruvan, Sweden (65°22’N; 18°38’E; hereafter mine C-78) and the gold mine Saattopora, which is located in Lapland of Northern Finland (67°47’N; 24°24’E; hereafter mine C-96; Figure S1). The Saattopora mine was closed in 1996. The mosses were sampled from a nearby pond at mine A, and a nearby stream at mines C-78 & C-96, and either received or were recently in contact with mine discharge water. The control site was groundwater-dependent spring ecosystems located in Finnish Lapland (67°23’N;24°25’E; hereafter control, respectively). The mosses from control site were sampled from the immediacy of the spring pool. We collected three replicates of moss samples per site on an average of 500 g wet weight. The samples were transported to the laboratory straight after sampling in the water of the sampling location. The sampling was carried out with the permission of landowners. Water quality, including pH, electrical conductivity (EC), dissolved oxygen (DO), and water temperature was measured in the field with a field meter (WTW Multi 350i meter) at the sampling time (Table S4). In the laboratory, the moss samples from each location were separated and subjected to surface sterilization, as described below, prior to freezing. Altogether, the microbiome data of the floating-hook moss contained 66 samples of both bacteria and fungi, the three mining sites included 48 samples of which 27 were separated as endophytes. Similarly, the control site included 18 samples of which nine were endophytes (more details in Table S5).

**Light microscopy**

Moss samples were cut to 3-mm pieces and fixed under vacuum in 4% paraformaldehyde (w/v), 0.1% glutaraldehyde (v/v), 20% glycerol (v/v), and 0.1 M sodium phosphate buffer (pH 7.4) for 2 h at 4°C. Using a cryomicrotome (Reichert-Jung 2800 Frigocut, Heidelberg, Germany), 15–25-μm sections were cut and mounted on microscopy slides with 0.1 M sodium phosphate buffer (pH 7.4) containing 10% glycerol (v/v). The sections of moss tissues were viewed with light microscope (Leica DM6 THUNDER Imager, Leica Microsystems, Wetzlar, Germany). Images were processed using the Leica Application Suite X software (LAS X ver. 3.8.1; Leica Microsystems, Wetzlar, Germany).

**Elemental and transmission electronic microscopy (TEM) analysis of floating-hook moss**

Cadmium (Cd), copper (Cu), lead (Pb), zinc (Zn), nickel (Ni), arsenic (As), and silicon (Si) from the moss biomass were measured with atomic absorption spectroscopy (AAS; Table S1), flame and graphite oven, specify the metals, among those investigated, were revealed using with a standard addition method to get rid of matrix effects and reference materials to cope with drift of the equipment. Three replicates for each site were quantified, and for the results, the mean, and the standard deviation for the four sampling sites were reported. In addition, as part of the HybArkt project [1] several other metals including reported Fe were measured from the moss tissue of control and mine A, and from the incoming water of mine A, by inductively coupled plasma mass spectrometry (Table S2). For the TEM analysis, the moss shoots from both the control site and mine A were fixed in 1% glutaraldehyde 4% formaldehyde mixture in 0.1 M phosphate buffer, post-fixed in 1% osmium tetroxide, dehydrated in acetone, and embedded in Epon LX 112 (Ladd Research Industries, Williston, Vermont, USA). Thin sections (70 nm) were cut with Leica Ultracut UCT ultramicrotome, collected on Butvar-coated copper grids, and stained using uranyl acetate and lead citrate. Grids without post-staining were also prepared. The fixed samples were processed also without using osmium tetroxide to detect metal particles. Sections on grids were examined with a Tecnai G2 Spirit 120 kV transmission electron microscope (FEI Europe, Eindhoven, The Netherlands) operated at 100 kV. The images were captured with Quemesa bottom-mounted CCD camera (Olympus Soft Imaging Solutions GMBH, Münster Germany) using Radius software (EMSIS GmbH, Münster, Germany). For the metal analysis, 5 nm of carbon was evaporated (Q150T ES, Quorum Technologies, UK) on the top of sections, and the analysis was performed using JEOL JEM-2200FS Scanning Transmission Electron Microscope (JEOL Ltd, Tokyo, Japan) equipped with Bright Field (BF) and High Angle Annular Dark Field (HAADF) detectors for STEM imaging, and JEOL Dry SD100GV EDS detector for the elemental analysis. The elemental state was determined using selected area electron diffraction (SAED). Energy-dispersive X-ray spectroscopy (EDS) data were analyzed using the JEOL Analysis Station Software.

**Surface sterilization and molecular analyses**

The microbiome was studied from the moss, the samples prior to surface sterilization containing both epiphytes and endophytes (hereafter named as moss bacteriome or mycobiome), and the surface-sterilized samples containing only endophytes (hereafter, endophytes). The moss material was pooled by collecting several moss shoots per sample, and the samples were used for DNA isolation. For the surface sterilization, mosses were first washed with tap water and dried gently. Then samples were washed with sterile Milli-Q water using a shaker at 200 rpm for 10 min. The mosses were surface sterilized with 0.5% sodium dichloroisocyanurate (NaDcc) for 3 min. Finally, the samples were rinsed twice in sterile Milli-Q water for 7 min and dried on sterilized paper to absorb excess water. Gentle shaking was implemented throughout the sterilization steps. The method was verified using plate culture validation.

DNA was extracted using Genomic DNA from plant protocol of NucleoSpin Plant II kit (Macherey-Nagel, Düren, Germany) with cell lysis step 2a (using Buffer PL1 and incubation at 65°C for 45 min). Amplification of the bacterial V4 region of the 16S rRNA gene was performed in triplicates using universal forward primer 515F (5′-GTG CCA GCM GCC GCG GTA A -3′) and 806R (5′- GGA CTA CHV GGG TWT CTA AT-3′) reverse primer [2].

The 25 µL 16S PCR mixture consisted of Phusion High-Fidelity DNA polymerase (Thermo Scientific, Waltham, USA), Phusion GC buffer, 0.2 mM dNTPs, 10 µg BSA, 0.4 µM of each 515F and 806R primer, and 30 ng of template DNA. To block the amplification of moss mitochondrion (mPNA) and plastid (pPNA) sequences, 1.2 µM of both mPNA (5′-GGC AAG TGT TCT TCG GA-3′) and pPNA (5′-GGC TCA ACC CTG GAC AG-3′) PCR clamps (PNA Bio, Newbury Park, CA, USA; [3]) were added to the reactions. The PCR conditions were as follows; initial denaturation of 98°C for 3 min, followed by 25 cycles of denaturation of 98°C for 20 s, PNA annealing of 78°C for 10 s, primer annealing of 50°C for 50 s, and extension at 72°C for 60 s.

To characterize the moss fungal communities, the internal transcribed spacer 2 (ITS2) region was amplified using ITS9F (5′- GAA CGC AGC RAA IIG YGA-3′; [4]) and ITS4R (5′- TCC TCC GCT TAT TGA TAT GC -3′; [5]) primers. The 25 µL ITS2 PCR mixture consisted of 2% DMSO, 0.4 µM of each ITS9F and ITS4R primer, while the rest of the reaction was identical to the 16S PCR reactions. The ITS2 PCR cycling conditions were 98°C for 3 min, 30 amplification cycles of 98°C for 10 s, 50°C for 20 s, and 72°C for 30 s, followed by a final extension step of 72°C for 7 min. After the amplification, sequencing libraries were generated from the pooled PCR products of the triplicate reactions and quality controlled following the protocol of the Earth Microbiome project [6]. The resulting amplicons for 16S and ITS2 were sequenced in separate runs using Illumina Miseq (PE300) system (Illumina, San Diego, CA, USA) at FIMM (Helsinki, Institute for Molecular Medicine Finland).

**Isolation and molecular identification of endophytes**

Surface-sterilized mosses were grown on potato dextrose agar (PDA) and Trypticase soy agar (TSA) for 2 weeks at 28°C. After subculturing and morphological characterization of pure cultures fungal endophytes were identified using molecular methods. Fungal hyphae were homogenized using liquid nitrogen and 100 mg was used for DNA extraction using NucleoSpin Plant II kit. PCR amplification was performed using 10−40 ng of extracted DNA as the template in a 20 μL reaction. Each reaction included 5× Phusion GC Buffer, 10 mM dNTPs, DMSO, 10 μM of each primer, and 0.025 U of Phusion High-Fidelity DNA polymerase. PCR was performed with an initial denaturation at 98 °C for 1 min, followed by 30 cycles at 98 °C for 10 s, 61 °C for 30 s, and 72 °C for 15 s, with a final extension at 72 °C for 5 min. The ITS region was amplified using the primers ITS1F (5′-CTT GGT CAT TTA GAG GAA GTA A-3′), ITS4R (5′-GGA AGT AAA AGT CGT AAC AAG G-3′; [5]), 58A2R (5′-CTG CGT TCT TCA TCG AT-3′;[7]) and fITS7 (5′-GTG ART CAT CGA ATC TTT GAA-3′;[4]). Purification and sequencing protocol with ABI 3730 DNA sequencing system (Thermo Fisher Scientific, Waltman, Massachusetts, USA) followed the method described by [8]. Raw sequence chromatograms were inspected and edited using BioEdit v7.2 [9]. Low quality regions were manually removed and primer sequences trimmed. Forward and reverse reads were aligned and assembled into consensus sequences, which were then used for species identification via BLASTn searches against the NCBI [10] and UNITE databases [11]. Species hypotheses from the UNITE database were used to support identification.

**Bioinformatics and statistical analyses**

The amplicon sequences were processed using the QIIME2 (v2021.2) next-generation microbiome bioinformatics pipeline [12]. Demultiplexed pair-end reads were processed, adapter-trimmed using q2-cutadapt plugin [13], and merged (truncate length 16S; forward 250 bp and reverse 210 bp, ITS; forward 240 bp and reverse 200 bp) using DADA2 [14]. The taxonomy was assigned to bacterial and fungal exact sequence variants (ESVs) using Naïve Bayesian pretrained feature‐classifier [15] against the SILVA 16S version 138 Gene Database [16] and the UNITE version 8 (qiime release 04.02.2020 dynamic files) database [17], respectively. Prior to further analyses, mitochondrial and chloroplast sequences were removed from the 16S rRNA dataset. The bacterial sequence run produced ~2 M reads. After filtering short reads (< 100 bp) and primers, the bacterial sequence data included 674,000 reads, while denoising reduced the read number to ~244,000. Of the reads, 58% were mitochondrial and chloroplast sequences, and 2.5% were unassigned ESVs. After trimming and denoising, the bacterial endophyte data included ~762,000 and ~294,000 reads, respectively. However, 93% of reads were mitochondrial and chloroplast sequences, and therefore bacterial endophyte data was not included in the further analyses. In total, 3369 ESVs were obtained for the bacteriome, of which 2873 were classified as bacterial. The fungal sequence run produced ~ 6 M reads, and data included ~ 2.7 M reads after trimming. Of the 370,000 denoised reads, 39% were unassigned ESVs. The fungal endophyte data consisted of ~ 2.6 M and ~ 155,000 reads after trimming and denoising, and of the denoised reads, only 3.2% were unassigned ESVs. We identified 1985 ESVs, of which 1656 were classified as fungi, while the endophytic sequence data consisted of 393 fungi and 511 ESVs. The closed mine C-96 data included only three endophyte replicates that after denoising consisted of < 50 reads, and therefore the mine C-96 was left out from further analyses of fungal endophytes. For further analyses fungus *Pezoloma ericae* was corrected to *Hyaloscypha hepaticola* [18].

The amplicon data was further processed with *phyloseq* [19] and *microeco* [20] packages in R statistical software [21]. ESV richness was not normally distributed, and therefore the data was tested using the Wilcoxon signed rank test. Prior to the taxonomic compositional analyses, the data was centered log-ratio (CLR) transformed with *microbiome* package [22]. The differences in taxonomic compositions among mining and control sites were examined using PERMANOVA with Aitchison dissimilarity coefficient on clr transformed relative abundance data [23]. PERMANOVA was run using *adonis* function in *vegan* package [24]. The difference in community composition was visualized with principal coordinate analysis (PCoA). To identify differently abundant taxa among the sites, we used DESeq2 [25] with *phyloseq_to_deseq2* extension. Prior to DESeq2 analysis, the explanatory data analysis was applied to reveal if one or more groups had much higher within-group variability than the others. Therefore, further analysis was applied for the data after splitting into pairs of groups. Bivariate comparisons were a priori made against the control. Core taxa that occurred in at least 90% of samples and that were shared between multiple groups were studied by Venn diagrams using *microbiome* and *eulerr* [26] packages.

**REFERENCES**

1. Postila, Heini, Elisangela Heiderscheidt, Anne Korhonen, Kaisa Lehosmaa, Ritva Nilivaara, Anna-Kaisa Ronkanen, et al. 2021. “Passiiviset Hybridipuhdistusratkaisut Arktisten Valumavesien Typen Ja Raskasmetallien Puhdistamiseen.” *HybArkt-Hankkeen Loppuraportti*. <http://hdl.handle.net/10138/324496>.

2. Parada, Alma E, David M Needham, Jed A. Fuhrman. 2016. “Every base matters: assessing small subunit rRNA primers for marine microbiomes with mock communities, time series and global field samples.” *Environmental Microbiology* 18: 1403–1414. <https://doi.org/10.1111/1462-2920.13023>.

3. Lundberg, Derek S, Scott Yourstone, Piotr Mieczkowski, Corbin D Jones, Jeffery L Dang. 2013. “Practical innovations for high-throughput amplicon sequencing.” *Nature Methods* 10: 999–1002. <https://doi.org/10.1038/nmeth.2634>.

4. Ihrmark, Katarina, Inga TM Bödeker, Karelyn Cruz-Martinez, Hanna Friberg, Ariana Kubartova, Jessica Schenck, et al. 2012. “New primers to amplify the fungal ITS2 region - evaluation by 454-sequencing of artificial and natural communities.” *FEMS Microbiology Ecology* 82: 666–677. <https://doi.org/10.1111/j.1574-6941.2012.01437.x>.

5. White, TJ, Tom Bruns, Steven Lee, John Taylor. 1990. “Amplification and direct sequencing of fungal ribosomal RNA genes for phylogenetics.” *PCR Protocols* 315-322. <https://doi.org/10.1016/B978-0-12-372180-8.50042-1>.

6. Thompson, Luke R, Jon G Sanders, Daniel McDonald, Amnon Amir, Joshua Ladau, Kenneth J. Locey, et al. 2017. “A communal catalogue reveals Earth’s multiscale microbial diversity.” *Nature* 551(7681):457-463. <https://doi.org/10.1038/nature24621>.

7. Martin, Kendall J, Paul T. Rygiewicz. 2005. “Fungal-specific PCR primers developed for analysis of the ITS region of environmental DNA extracts.” *BMC Microbiology* 5(1):28. <https://doi.org/10.1186/1471-2180-5-28>.

8. Nguyen, Minh-Phuong, Kaisa Lehosmaa, Françoise Martz, Janne J. Koskimäki, Anna Maria Pirttilä, Hely Häggman. 2021.” Host species shape the community structure of culturable endophytes in fruits of wild berry species (*Vaccinium myrtillus* L., *Empetrum nigrum* L. and *Vaccinium vitis-idaea* L.).” *FEMS Microbiology Ecology* 97(8):fiab097. <https://doi.org/10.1093/femsec/fiab097>.

9. Hall TA. 1999. “BioEdit: A User-Friendly Biological Sequence Alignment Editor and Analysis Program for Windows 95/98/NT.” *Nucleic Acids Symposium Series* 41:95-98.

10. Altschul, Stephen F, Warren Gish, Webb Miller, Eugene W Myers, David J. Lipman. 1990. “Basic local alignment search tool.” *Journal of Molecular Biology* 215(3):403-410. <https://doi.org/10.1016/S0022-2836(05)80360-2>.

11. Abarenkov, Kessy, R Henrik Nilsson, Karl-Henrik Larsson, Andy FS Taylor, Tom W May, Tobias Gulberg Frøslev, et al. 2024. “The UNITE database for molecular identification and taxonomic communication of fungi and other eukaryotes: sequences, taxa and classifications reconsidered.” *Nucleic Acids Research* 52(D1):D791-D797. <https://doi.org/10.1093/nar/gkad1039>.

12. Bolyen, Evan, Jai Ram Rideout, Matthew R Dillon, Nicholas A Bokulich, Christian C Abnet, Gabriel A Al-Ghalith, et al. 2019. “Reproducible, interactive, scalable and extensible microbiome data science using QIIME 2.” *Nature Biotechnology* 37(8):852-857. <https://doi.org/10.1038/s41587-019-0209-9>.

13. Martin, Marcel. 2011. “Cutadapt removes adapter sequences from high-throughput sequencing reads.” *EMBnet Journal* 17(1):10. <https://doi.org/10.14806/ej.17.1.200>.

14. Callahan, Benjamin J, Paul J McMurdie, Michael J Rosen, Andrew W Han, Amy Jo Johnson, Susan P Holmes. 2016. “DADA2: High-resolution sample inference from Illumina amplicon data.” *Nature Methods* 13(7):581-583. <https://doi.org/10.1038/nmeth.3869>.

15. Bokulich, Nicholas A, Benjamin D Kaehler, Jai Ram Rideout, Matthew Dillon, Evan Bolyen, Rob Knight, et al. 2018. “Optimizing taxonomic classification of marker-gene amplicon sequences with QIIME 2’s q2-feature-classifier plugin.” *Microbiome* 6(1):90. <https://doi.org/10.1186/s40168-018-0470-z>.

16. Quast, Christian, Elmar Pruesse, Pelin Yilmaz, Jan Gerken, Timmy Schweer, Pablo Yarza, et al. 2013. “The SILVA ribosomal RNA gene database project: Improved data processing and web-based tools.” *Nucleic Acids Research* 41(D1):590-596. <https://doi.org/10.1093/nar/gks1219>.

17. Kõljalg, Urmas, Henrik R Nilsson, Dmitry Schigel, Leho Tedersoo, Karl-Henrik Larsson, Tom W May, et al. 2020. “The taxon hypothesis paradigm—on the unambiguous detection and communication of taxa.” *Microorganisms* 8(12):1910. <https://doi.org/10.3390/microorganisms8121910>.

18. Fehrer, J, M Réblová, V Bambasová, M Vohník. 2019. “The root-symbiotic *Rhizoscyphus ericae* aggregate and *Hyaloscypha* (*Leotiomycetes*) are congeneric: Phylogenetic and experimental evidence.” *Studies in Mycology* 92(1):195-225. <https://doi.org/10.1016/j.simyco.2018.10.004>.

19. McMurdie, Paul J, Susan Holmes. 2013. “phyloseq: An R package for reproducible interactive analysis and graphics of microbiome census data.” *PLoS One* 8(4):e61217. <https://doi.org/10.1371/journal.pone.0061217>.

20. Liu, Chi, Yaoming Cui, Xiangzhen Li, Minjie Yao. 2021. “*microeco*: an R package for data mining in microbial community ecology.” *FEMS Microbiology Ecology* 97(2):fiaa255. <https://doi.org/10.1093/femsec/fiaa255>.

21. R Core Team. 2022. “R: A Language and Environment for Statistical Computing.” Vienna, Austria: R Foundation for Statistical Computing.

22. Lahti Leo, Sudarshan Shetty. 2020. “Tools for microbiome analysis in R”. Published online.

23. Gloor, Gregory B, Jean M Macklaim, Vera Pawlowsky-Glahn, Juan J Egozcue. 2017. “Microbiome datasets are compositional: and this ss not optional.” *Frontiers in Microbiology* 8:2224. <https://doi.org/10.3389/fmicb.2017.02224>.

24. Oksanen, Jari, FG Blanchet, M Friendly, R Kindt, P Legendre, D McGlinn, et al. 2019. “Package ‘vegan.’” *Community ecology package, version*.

25. Love, Michael I, Wolfgang Huber, Simon Anders. 2014. “Moderated estimation of fold change and dispersion for RNA-seq data with DESeq2.” *Genome Biology* 15(12):550. <https://doi.org/10.1186/s13059-014-0550-8>.

26. Larsson Johan. 2022. “eulerr: Area-Proportional Euler and Venn Diagrams with Ellipses.” Published online.

**Figure S1** **Map of sampling sites in Finland and Sweden**. Control = site with relatively low metal concentrations, mine A = active mine, and mines C-78 and C-96 are closed mines.

**Figure S2** **Transmission electron microscopy (TEM) of an unstained mine A sample**. TEM and selected area electron diffraction (HAADF-STEM) images of moss tissue from unstained sample of mine A (A), line scan image of the precipitated metals (area studied dashed square of (A)) inside the moss cell (B). (C−F) images illustrate the distribution of detected metals (C) silica, (D) iron (10.9 mass%), (E) chloride (2 mass%), and (F) oxygen (14.9 mass%). Abbreviations in figure A are, C= cytoplasm and IC= intercellular space. Scale bars (A) 1 µm and (C−F) 2 µm.

**Figure S3 Transmission electron microscopy (TEM) of a stained and unstained control sample.** (A−D) Transmission electron microscopy (TEM) images of *W. fluitans* tissue from control site. (A and B) The images of unstained samples and (C and D) samples stained with uranyl acetate and lead citrate. Yellow arrows + Fh= fungal hyphae colonizing the gametophyte cell walls, yellow dotted line + Mc= a microbial cell inside the gametophyte cell, Gc= gametophyte cell, Ext= exterior, and Ic= intercellular space. Scale bars (A and B) 1 µm and (C and D) 2 µm.
